# Valine/isoleucine variants drive selective pressure in the VP1 sequence of EV-A71 enteroviruses

**DOI:** 10.1186/s12879-017-2427-4

**Published:** 2017-05-08

**Authors:** Nghia Ngu Duy, Le Thi Thanh Huong, Patrice Ravel, Le Thi Song Huong, Ankit Dwivedi, October Michael Sessions, Yan’An Hou, Robert Chua, Guilhem Kister, Aneta Afelt, Catherine Moulia, Duane J. Gubler, Vu Dinh Thiem, Nguyen Thi Hien Thanh, Christian Devaux, Tran Nhu Duong, Nguyen Tran Hien, Emmanuel Cornillot, Laurent Gavotte, Roger Frutos

**Affiliations:** 1 National Institute of Hygiene and Epidemiology, 1 Pho Yersin Street, Hanoi, 10000 Vietnam; 2 University of Montpellier, ISEM, CC063, Place E. Bataillon, 34095 Montpellier Cedex 5, France; 3 Cirad, UMR 17, Intertryp, TA-A17/G, Campus International de Baillarguet, 34398 Montpellier Cedex 5, France; 4 Institut de Recherche en Cancérologie de Montpellier (U1194), Campus Val d’Aurelle, 34298 Montpellier Cedex 5, France; 5 Hai Phong Preventive Medicine Center, Hai Phong city, Vietnam; 6 Institut de Biologie Computationnelle, MMVE, La Galera, CC6005, 95 rue de la Galera, 34095 Montpellier, France; 7 DUKE-NUS Graduate Medical School, 8 College Road, Singapore, Singapore; 8 Faculty of Pharmacy, University of Montpellier, 15 av Charles Flahault, BP14491, 34093 Montpellier Cedex 5, France; 9 Faculty of Geography and Regional Studies, University of Warsaw, Krakowskie Przedmiescie 26/28, 00-927 Warsaw, Poland; 10 Institut de Recherche pour le Développement (IRD), Le Sextant, 44, bd de Dunkerque, CS 90009, 13572 Marseille cedex 02, France; 11 Université de Montpellier, IES – Institut d’Electronique et des Systèmes, UMR 5214, CNRS-UM, 860 rue St. Priest, Bt. 5, 34095 Montpellier, France

**Keywords:** VP1, HFMD, Enterovirus, EV-A71, Vietnam

## Abstract

**Background:**

In 2011–2012, Northern Vietnam experienced its first large scale hand foot and mouth disease (HFMD) epidemic. In 2011, a major HFMD epidemic was also reported in South Vietnam with fatal cases. This 2011–2012 outbreak was the first one to occur in North Vietnam providing grounds to study the etiology, origin and dynamic of the disease. We report here the analysis of the VP1 gene of strains isolated throughout North Vietnam during the 2011–2012 outbreak and before.

**Methods:**

The VP1 gene of 106 EV-A71 isolates from North Vietnam and 2 from Central Vietnam were sequenced. Sequence alignments were analyzed at the nucleic acid and protein level. Gene polymorphism was also analyzed. A Factorial Correspondence Analysis was performed to correlate amino acid mutations with clinical parameters.

**Results:**

The sequences were distributed into four phylogenetic clusters. Three clusters corresponded to the subgenogroup C4 and the last one corresponded to the subgenogroup C5. Each cluster displayed different polymorphism characteristics. Proteins were highly conserved but three sites bearing only Isoleucine (I) or Valine (V) were characterized. The isoleucine/valine variability matched the clusters. Spatiotemporal analysis of the I/V variants showed that all variants which emerged in 2011 and then in 2012 were not the same but were all present in the region prior to the 2011–2012 outbreak. Some correlation was found between certain I/V variants and ethnicity and severity.

**Conclusions:**

The 2011–2012 outbreak was not caused by an exogenous strain coming from South Vietnam or elsewhere but by strains already present and circulating at low level in North Vietnam. However, what triggered the outbreak remains unclear. A selective pressure is applied on I/V variants which matches the genetic clusters. I/V variants were shown on other viruses to correlate with pathogenicity. This should be investigated in EV-A71. I/V variants are an easy and efficient way to survey and identify circulating EV-A71 strains.

**Electronic supplementary material:**

The online version of this article (doi:10.1186/s12879-017-2427-4) contains supplementary material, which is available to authorized users.

## Background

Hand, foot and mouth disease (HFMD) is an acute febrile illness in children with a papulovesicular skin rash at the palms or soles of the feet, or both. Presentation can be with or without inclusion of mouth ulcers. Although the disease is usually mild and self-limiting, in some cases HFMD can result in severe complications such as encephalitis, aseptic meningitis, pulmonary edema, myocarditis, and death [29]. HFMD is caused by members of Human Enterovirus A, a family of picornaviridae which includes Coxsackievirus A (CV-A) and Human Enterovirus 71 (EV-A71) [3, 7]. The EV-A71 viruses are genetically related to CV-A; indeed, it has been suggested that these viruses may have diverged as recently as the 1940s [27]. Both EV-A71 and CV-A infections have been associated with severe HFMD in young children, sometimes resulting in death [2, 21, 33].

Enteroviruses are characterized by the presence of 4 structural proteins, VP4 being the internal capsid protein and VP1, VP2 and VP3 making the three external capsid proteins [6]. VP1 is the most external and is the main component of the canyon on the surface of picornaviruses. VP1 is involved in viral pathogenicity, receptor binding and immune modulation of EV-A71 [13, 31]. Differences in EV-A71 strains might contribute to the different severity of the disease [18, 24] and virulence determinants have been identified in the VP1 protein such as residues G/Q/R at position VP1–145, E at VP1–164 [5, 12, 16]. VP1 is used to classify enteroviruses. Based on the VP1 gene, EV-A71 is classified into three independent genogroups: A, B, and C. The EV-A71 B and C genogroups are each further subdivided into five subgenogoups, B1 to B5 and C1 to C5 [4].

Although EV-A71 was isolated for the first time in Vietnam in 2003, the first outbreak of HFMD was not reported in the southern provinces until 2005. The 2005 outbreak was associated with EV-A71 C1, C4 and C5 genotypes and Coxsackievirus A16 [14, 28]. In 2011, a major HFMD epidemic was reported in South Vietnam with fatal cases reported [19]. This 2011–2012 outbreak was the first one to occur in North Vietnam providing grounds to study the etiology, origin and dynamic of the disease. We report here the analysis of the VP1 gene of strains isolated throughout North Vietnam during the 2011–2012 outbreak.

## Methods

### Epidemiological information and source of specimens

All HFMD cases in Northern provinces were reported to the National Institute of Hygiene and Epidemiology (NIHE) through the national communicable disease surveillance system since 2011. HFMD patients that reported to health centers or hospitals were diagnosed and classified in 4 severity levels (Additional file 1: Table S1). The evaluation of the disease was performed according to the guidelines specifically published by the Vietnamese Ministry of Health which are based on WHO and Taiwanese guidelines [11, 29]. A hundred and eight EV-A71 throat swabs from North Vietnam and 2 from Central Vietnam were collected from 2003 to 2012.

### Sampling

Ninety four samples were obtained from 94 different hospitalized patients diagnosed with EV A71 HFMD in 19 out of 28 provinces in North Vietnam in 2011 and 2012 and stored at −80 °C. Fourteen reference samples obtained from previous cases of EV A71 HFMD between 2003 and 2010 in seven provinces in North Vietnam and two provinces in Central Vietnam were included in the study (Table 1). All samples were sent to the Enterovirus Laboratory of NIHE for etiological assays. Enterovirus-positive and EV-A71-positive samples were identified according to Nix et al. [20] using *SO* (SO224/SO222), *AN* (AN88/AN89) and *MAS* (MAS01S/MAS02A) [22] primer sets. Viral RNA was directly extracted from throat swab using QIAamp® Viral RNA Mini Kit (Qiagen, Valencia, USA). The cDNA was prepared using the GoScript™ Reverse Transcriptase kit from Promega. Seminested RT-PCR was conducted as described by Nix et al. [20]. The cDNA was first synthesized from the RNA for 10 min at 25 °C and followed by synthesis of the second strand at 42 °C for 50 min, 72 °C for 15 min using primers AN32, AN33, AN34 and AN35 [20]. PCR was done as described by Nix et al. [20] with 40 cycles of amplification (95 °C for 30 s, 42 °C for 30 s and, 60 °C for 45 s). One microliter of the first PCR was used a second seminested amplification for 40 amplification cycles of 95 °C for 30 s, 60 °C for 20 s, and 72 °C for 15 s. Sequencing was performed with the Sanger method using the is Bigdye Terminator V3.1 cycle sequencing kit from Applied Biosystems in an ABI sequencer 3130.

**Table 1:** Characteristics of the isolated EV-A71 strains

Strain	Age (month)	Gender	Province	Ethnicity	Time from onset to collection (days)	Date	Severity^f^	V/I Type	Subgenogroup	Accession number
2003019	12	NA^a^	Hà Nội	NA	4	03/2003	NA	VIV	C4	KX906272
2005184	0	NA	Quảng Nam	NA	2	05/2005	NA	VVI	C5	KX906332
2006023	0	NA	Phú Yên	NA	3	04/2006	NA	VIV	C4	KX906278
2007015	24	NA	Hà Nam	NA	3	01/2007	NA	IVI	C5	KX906365
2007037	24	NA	Yên Bái	NA	1	03/2007	NA	IVI	C5	KX906264
2007041	144	NA	Cao Bằng	NA	2	03/2007	NA	VII	C4	KX906262
2007053	30	NA	Hải Phòng	NA	3	04/2007	NA	VII	C4	KX906261
2008014	24	1^b^	Nam Định	NA	2	05/2008	NA	VII	C4	KX906263
2008017	30	1	Ninh Bình	NA	3	05/2008	NA	VII	C4	KX906267
2008021	30	2^c^	Ninh Bình	NA	2	05/2008	NA	IVI	C5	KX906299
2008022	24	2	Ninh Bình	NA	0^g^	05/2008	NA	IIV	C4	KX906273
2008044	30	1	Ninh Bình	NA	2	06/2008	NA	IVI	C5	KX906300
2008065	30	1	Hải Phòng	1^d^	3	06/2008	1	IVI	C5	KX906345
2010002	19	2	Bắc Kạn	NA	3	05/2010	NA	IVI	C5	KX906358
2011011	20	2	Hòa Bình	2^e^	1	06/2011	1	VII	C4	KX906301
2011020	24	2	Hòa Bình	2	1	06/2011	1	VII	C4	KX906289
2011022	28	1	Hòa Bình	2	1	06/2011	1	VII	C4	KX906315
2011031	72	2	Hà Nội	1	3	06/2011	2a	VIV	C4	KX906338
2011033	26	1	Hòa Bình	2	1	08/2011	1	VII	C4	KX906274
2011034	48	2	Hòa Bình	1	1	06/2011	1	VIV	C4	KX906292
2011047	21	1	Sơn La	NA	4	07/2011	1	VII	C4	KX906269
2011048	22	1	Sơn La	NA	4	07/2011	2a	VIV	C4	KX906352
2011060	21	2	Thanh Hóa	1	3	06/2011	2a	VVV	C4	KX906265
2011063	24	1	Hòa Bình	1	2	07/2011	1	VII	C4	KX906290
2011095	19	1	Hòa Bình	2	1	07/2011	1	VII	C4	KX906337
2011096	12	2	Hòa Bình	NA	3	07/2011	1	VII	C4	KX906308
2011097	7	1	Hòa Bình	1	6	09/2011	1	VII	C4	KX906335
2011117	42	1	Thanh Hóa	1	3	07/2011	2a	IIV	C4	KX906350
2011123	12	2	Hòa Bình	2	0	07/2011	1	VIV	C4	KX906281
2011124	9	1	Hòa Bình	2	1	07/2011	1	VII	C4	KX906329
2011125	13	1	Hòa Bình	NA	1	07/2011	1	VII	C4	KX906368
2011158	11	2	Hòa Bình	NA	2	08/2011	1	IVI	C5	KX906309
2011161	21	1	Hòa Bình	2	0	07/2011	1	VII	C4	KX906296
2011165	22	1	Hòa Bình	1	1	08/2011	1	VII	C4	KX906349
2011278	21	1	Nam Định	1	2	08/2011	1	VVV	C4	KX906310
2011282	23	1	Nam Định	1	0	08/2011	1	IIV	C4	KX906343
2011340	2	1	Lào Cai	2	1	08/2011	2a	VII	C4	KX906293
2011488	60	2	Hòa Bình	NA	2	09/2011	1	VII	C4	KX906319
2011490	38	1	Hòa Bình	2	1	09/2011	1	VII	C4	KX906276
2011521	43	2	Hà Nội	1	3	09/2011	1	VVV	C4	KX906305
2011571	32	1	Hòa Bình	2	1	09/2011	1	VIV	C4	KX906331
2011573	12	1	Hòa Bình	NA	1	09/2011	1	VII	C4	KX906307
2011575	12	1	Hòa Bình	2	4	09/2011	1	VIV	C4	KX906341
2011579	46	1	Hà Nội	1	2	09/2011	1	IIV	C4	KX906277
2011586	20	2	Hà Nội	1	3	09/2011	2a	IIV	C4	KX906366
2011598	26	1	Thanh Hóa	2	1	09/2011	1	VII	C4	KX906339
2011600	51	1	Bắc Kạn	1	7	10/2011	2a	VIV	C4	KX906355
2011647	22	2	Thanh Hóa	1	8	10/2011	2a	VII	C4	KX906268
2011662	11	1	Hà Nội	1	2	10/2011	1	IIV	C4	KX906297
2011664	16	2	Hà Nội	1	1	10/2011	2a	IIV	C4	KX906325
2011665	11	2	Hà Nội	1	3	10/2011	1	IIV	C4	KX906266
2011676	4	1	Điện Biên	2	3	10/2011	1	IIV	C4	KX906271
2011677	13	1	Điện Biên	1	3	10/2011	1	IIV	C4	KX906322
2011679	12	1	Thanh Hóa	NA	4	10/2011	1	IIV	C4	KX906270
2011754	8	1	Hòa Bình	NA	10	10/2011	1	IIV	C4	KX906334
2011799	12	1	Thanh Hóa	1	2	10/2011	2b	VII	C4	KX906275
2011805	9	2	Thanh Hóa	2	3	10/2011	2a	VII	C4	KX906286
2011816	22	1	Điện Biên	2	8	10/2011	2a	IIV	C4	KX906357
2011823	32	2	Tuyên Quang	1	10	04/2011	1	IIV	C4	KX906364
2011835	18	1	Hà Nội	1	2	11/2011	1	VII	C4	KX906313
2011840	15	2	Hà Nội	1	3	11/2011	2a	VII	C4	KX906340
2011866	26	2	Bắc Ninh	1	2	11/2011	1	IIV	C4	KX906354
2011868	52	2	Bắc Kạn	1	5	11/2011	1	VIV	C4	KX906330
2011872	13	2	Điện Biên	1	2	11/2011	2a	IIV	C4	KX906347
2011881	26	2	Hải Phòng	1	3	11/2011	2b	VII	C4	KX906360
2011882	25	1	Hải Phòng	1	6	11/2011	2b	IIV	C4	KX906287
2011888	25	2	Hải Phòng	1	4	11/2011	2b	VII	C4	KX906333
2011891	26	1	Thanh Hóa	1	3	11/2011	1	VII	C4	KX906361
2011894	20	1	Hải Phòng	1	5	11/2011	2a	IIV	C4	KX906312
2011896	17	2	Hải Phòng	1	5	11/2011	2b	IIV	C4	KX906336
2011897	11	1	Hải Phòng	1	6	11/2011	2b	VIV	C4	KX906317
2011925	13	2	Hải Phòng	1	1	11/2011	2b	IIV	C4	KX906363
2011927	41	2	Hòa Bình	1	3	11/2011	1	VII	C4	KX906283
2011956	64	2	Tuyên Quang	2	13	11/2011	3	VII	C4	KX906304
2011958	26	1	Tuyên Quang	2	7	11/2011	3	VII	C4	KX906323
2011970	32	1	Hà Nội	1	1	11/2011	2a	IIV	C4	KX906298
2011984	13	1	Hải Phòng	1	5	12/2011	2a	IIV	C4	KX906327
2012018	18	2	Hải Dương	1	3	02/2012	1	IVI	C5	KX906295
2012019	18	1	Hải Dương	1	1	02/2012	1	VIV	C4	KX906353
2012053	12	1	Lào Cai	1	3	02/2012	2a	VIV	C4	KX906359
2012095	20	1	Bắc Giang	1	1	03/2012	2a	IIV	C4	KX906367
2012114	20	1	Lào Cai	1	1	03/2012	2a	VII	C4	KX906303
2012117	20	1	Lào Cai	2	2	03/2012	1	IVI	C5	KX906280
2012126	11	1	Hải Phòng	1	5	03/2012	2b	IIV	C4	KX906346
2012151	13	1	Hải Dương	1	2	03/2012	1	VII	C4	KX906320
2012159	36	2	Phú Thọ	1	3	03/2012	2a	VIV	C4	KX906314
2012161	16	1	Phú Thọ	1	4	03/2012	2a	VIV	C4	KX906302
2012164	27	1	Phú Thọ	2	2	02/2012	1	IIV	C4	KX906306
2012165	54	1	Phú Thọ	2	0	02/2012	1	IIV	C4	KX906362
2012166	14	1	Phú Thọ	1	5	03/2012	1	VIV	C4	KX906356
2012169	5	2	Phú Thọ	1	3	03/2012	1	IVI	C5	KX906294
2012189	15	1	Hải Dương	NA	5	03/2012	1	VIV	C4	KX906285
2012225	7	1	Hà Nội	1	5	03/2012	3	VIV	C4	KX906348
2012233	40	1	Bắc Kạn	2	5	03/2012	1	IIV	C4	KX906351
2012237	84	2	Bắc Giang	1	3	03/2012	2b	IVI	C5	KX906288
2012260	25	1	Thanh Hóa	1	2	03/2012	1	VIV	C4	KX906311
2012262	30	1	Thanh Hóa	NA	3	03/2012	NA	VIV	C4	KX906328
2012264	9	1	Thanh Hóa	1	3	03/2012	1	IVI	C5	KX906284
2012271	34	1	Ninh Bình	1	1	03/2012	1	IVI	C5	KX906279
2012284	44	2	Hải Phòng	1	2	03/2012	2b	IVI	C5	KX906282
2012296	24	2	Sơn La	NA	2	03/2012	NA	VII	C4	KX906344
2012297	22	1	Tuyên Quang	1	13	03/2012	1	VII	C4	KX906342
2012300	28	1	Tuyên Quang	1	9	03/2012	1	VIV	C4	KX906326
2012319	57	2	Bắc Kạn	2	0	03/2012	1	VIV	C4	KX906318
2012353	17	2	Hà Nội	1	3	04/2012	1	VII	C4	KX906316
2012387	17	2	Lai Châu	1	3	04/2012	2a	IVI	C5	KX906291
20111000	14	2	Hải Phòng	1	3	12/2011	2b	IIV	C4	KX906324
20111003	19	1	Hải Phòng	1	5	12/2011	2b	VIV	C4	KX906321

^a)^NA: Not Available

^b)^Gender 1 = Male

^c)^Gender 2 = Female

^d)^Ethnicity 1 = Main Vietnamese ethnic group

^e)^Ethnicity 2 = Hmong ethnic minority

^f)^Severity is classified according to five scores: 1, 2a, 2b, 3 and 4.1 and 2a are mild cases and 2b to 4 are severe cases. The description of each stage is given in Additional file 1: Table S1

^g)^“0” means that admission occurred the same day as onset

### Sequence analyses

Sequences were deposited in GenBank and accession numbers are provided in Table 1. The VP1 genetic sequences were aligned in Seaview 4.6 [10] using Muscle algorithm [9]. Best-fitting evolutionary models were determined by JModelTest 2.1 [8] or by ProtTest 2.4 [1] using the corrected version of the Akaike Information Criterion (AICc). The phylogeny of VP1 was performed by Maximum Likelihood (ML) inference using the model GTR + G + F with Seaview 4.6 [10]. The robustness of nodes was assessed with 500 bootstrap replicates. ML analysis of the amino acid sequences was performed using the model JTT + I + G with Seaview 4.6 [10]. The robustness of nodes was assessed with 500 bootstrap replicates. Sequence polymorphism was investigated using the DnaSP 5.10.01 package [17]. Amino acid numbering was done with respect to full length polyprotein using the sequence HQ129932.1 [16] as a reference.

### Bias and ethics

Training session on HFMD cases definition and reporting were organized for the staff of the routine surveillance system to enhance quality and consistency of case report. This work was conducted following the requirements of the Vietnamese Ministry of Health and under the Law of Communicable Diseases Prevention and Control passed in 2007.

### Factorial correspondence analysis

A factorial correspondence analysis (FCA) was performed using XLSTAT software (Addinsoft®). Variables considered were: amino acid profiles on positions 151, 164 and 186 (V/I), respectively in this work (249, 262 and 284 on the full length VP1 protein), severity level, ethnicity, age of patients, and patient location. The best descriptive axes were retained, explaining 34% of the data spread. Potential correlations were confirmed by a test of contingency using Statview 5.0 software (SAS Institute Inc.)

### Spatio-temporal analysis

Administration data were obtained from GADM database of Global Administrative Areas (version 2.8, November 2015). Spatial analyses were conducted with Quantum GIS, version 2.8.2. All spatial data are in the WGS 84 coordinate system.

## Results

### Clinical and epidemiological features

Data are shown in Table 1. Patients age ranged from 2 months to 12 years old (median at 1.8 years, IQR of 1.5 years). 102/106 (96.23%) patients were under 5. The age-specific incidence highest in the 1–2 years age group (44 cases, 41.51%) and remained very low for older children. The lowest incidence was observed in infants under 6 months (2.83%) and children above 10 years old (0.94%). Patients came from all parts of the region including mountainous, rural and urban areas. Out of 83 cases, 59 (71.08%) belonged to main Vietnamese ethnicity (Ethnicity 1) while the rest of patients belonged to the minority Hmong ethnicity (Ethnicity 2) (Table 1). All severity levels were reported for the patients. Mild forms (severity level 1) made the majority of cases (57 cases, 61.29%) while 15 patients displayed severe symptoms (16.13%). Among this group, 3 patients displayed a severity score of 3. No case with the highest level of 4 was recorded. Moderate forms of HFMD were found in 21 patients (22.58). (Table 1).

### VP1 phylogeny and population structure

**Fig. 1:**
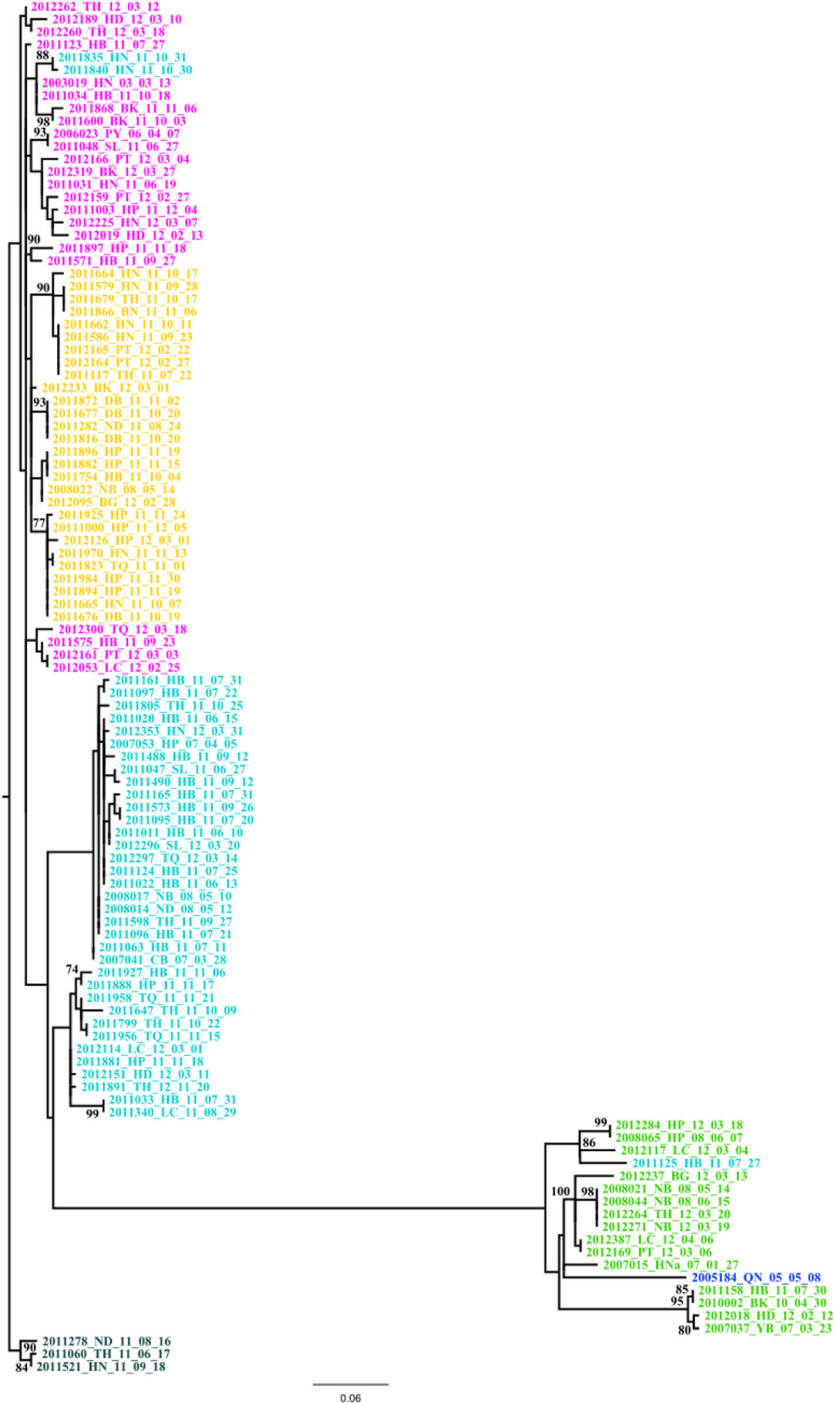
Phylogenetic analysis of partial VP1 sequences. **a** Phylogenetic analysis of the nucleic acid sequences.Tree was designed using Maximum Likelihood. Color code: *Black*: VVV; *Light blue*: VII; *Yellow*: IIV; *Purple*: VIV; *Dark blue*: VVI

**Fig. 2:**
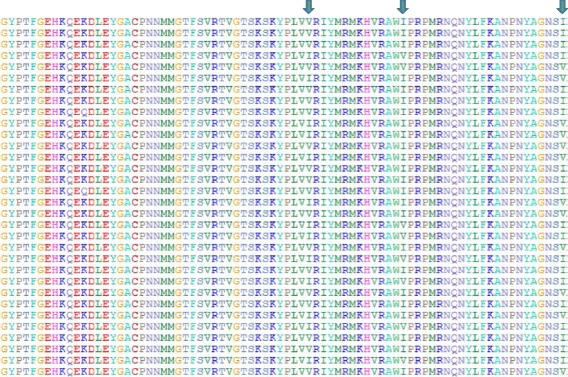
Multiple alignment of VP1 proteins. The three sites analyzed in this work are marked by arrows

Phylogenetic analysis of the VP1 gene sequences indicated the presence of four clusters (Fig. 1). Cluster 1, the closest to the root, was the main one, comprising 56 sequences and structured into several subclusters characterized by low bootstrap values. Cluster 2 and cluster 3 comprised respectively 23 and 12 sequences and segregated from cluster 1 to which they were separated with a low bootstrap of 30. The last cluster, cluster 4, comprising 17 sequences was characterized by a high bootstrap value (100) and was a derivate from cluster 3. A similar tree topology was observed when using protein alignments (Data not shown). With respect to the current genogroup classification, clusters 1, 2 and 3 belonged to the subgenogroup C4 whereas cluster 4 belonged to the subgenogroup C5 (Table 1). The VP1 protein was characterized by a high rate of conservation. However, three sites displayed a consistent Valine (V) / Isoleucine (I) variation (Fig. 2). These sites, sites A, B and C were located in the VP1 protein at position 814, 827 and 849 of the polyprotein. Six types of I/V variants were observed when considering the amino acids at sites A, B and C: VVI (1 sequence), IVI (15 sequences), IIV (28 sequences), VII (38 sequences), VIV (23 sequences) and VVV (3 sequences) (Table 1). When attributing a color code for each I/V variant population and applying it to the phylogenetic tree a clear overlap with the previously detected clusters was observed (Fig. 1). Cluster 4 overlapped with the IVI population while clusters 2 and 3 comprised the VII variants. The VVV, IVV and VIV variants all corresponded to cluster 1. However they did not mix and each one corresponded to a specific subcluster. The VVI variant derived from the cluster 4 / IVI group. Three exceptions were found, with VII variants present in clusters 1 and 4. With respect to phylogeny, the I/V variant closest to the root is VVV, the VIV population emerged from this group and gave rise in turn to the IIV group. The VII groups, derived from the VIV group with first cluster 2 from which cluster 3 evolved. The well separated cluster 4/IVI variants evolved from cluster 3. This overlap between clusters and I/V populations was even stronger at the protein level since almost all the variability was borne by the I/V mutations, the rest of the protein being highly conserved. Groups VII, IIV, VVV and VIV belonged to the subgenogroup C4 whereas groups IVI and VVI belonged to the subgenogroup C5.

### Nucleic acid polymorphism

When considering the polymorphism of the various clusters identified, very different traits were observed (Table 2). Cluster 1 was polymorphic (θ = 14.58) but with a 2.5 times more parsimony informative sites than singletons and 10-times more synonymous mutations than non-synonymous ones, suggesting that it is not a recent polymorphism or expanding population. The Ka/Ks ratio was also characterized by a low value. Conversely, cluster 2 displayed a very low level of polymorphism with a q of 4.06 and a low number of mutations η (η = 15). The Ka/Ks was slightly higher at 0.107. This is suggesting the existence of a bottleneck at the origin of cluster 2. Cluster 3 which originated from cluster 2 was more polymorphic with a slightly increasing θ (θ = 6.92) and number of mutations η (η = 19). Only synonymous mutations were observed resulting in turn in a very low Ka/Ks ratio of 0.029. Cluster 4 was on the other hand displaying a very high polymorphism with a η of 113 for 109 and a very high level of synonymous mutations (105 out of 110) suggesting a strong negative selection acting on a mutating population. As a consequence, the Ka/Ks ratio was also very low at 0.011.

**Table 2:** Polymorphism and divergence data

		N	Hp	Nt	S	η	Pa	Si	θ	MC	Na	Ns	Ka/Ks
Cluster 1	56	43	561	67	70	48	19	14.58	64	6	58	0.053	
Cluster 2	23	15	561	15	15	9	6	4.06	15	6	9	0.107	
Cluster 3	12	10	561	19	19	11	8	6.92	16	0	16	0.029	
Cluster 4	17	13	561	109	113	57	52	32.24	110	5	105	0.011	
Total		108	81	561	148	162	128	20	28.16	147	9	138	0.019

*N* Number of sequences

*Hp* Number of haplotypes

*Nt* Sequence size in nucleotides

*S* Number of mutated sites

*η* Number of mutations

*Pa* Number of parsimony informative sites

*Si* Number of singletons

*θ* Number segregating sites (per sequence from S)

*MC* Number of mutated codons

*Na* Number of non-synonymous mutations

*Ns* Number of synonymous mutations

*Ka/Ks* Ka/Ks ratio

### Distribution of mutations and correlation analysis

**Fig. 3:**
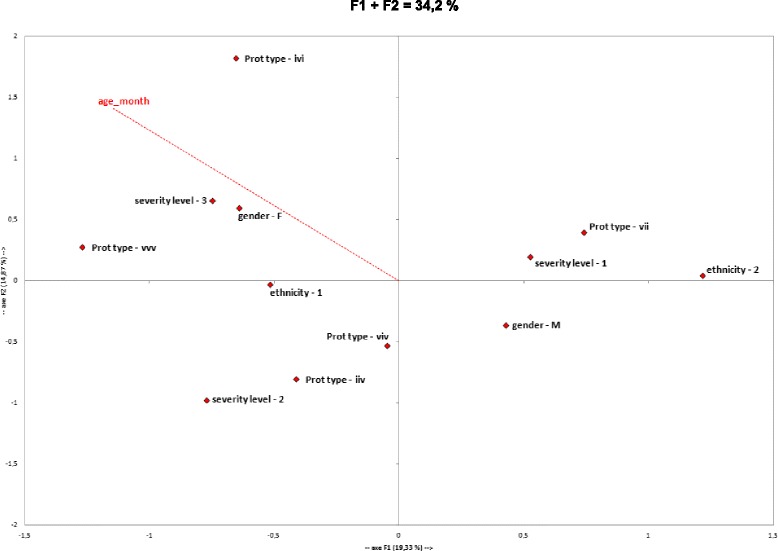
Factorial correspondence analysis. Variables analyzed were: amino acid profiles on positions 151, 164 and 186 (V/I), respectively in this work (249, 262 and 284 on the full length VP1 protein), severity level, ethnicity, age of patients, and patient location

The correlation analysis indicated a partial structuration of cases (34% of dispersion explained) on different parameters (Fig. 3). The VVI variant was not included in the analysis because all information was not available. The analysis was therefore conducted on only five variants, i.e. IIV, VII, IVI, VIV and VVV. Severity of HFMD seemed to correlate with the age of patient (*p* = 0.011) and the highest severity level was not observed above 11-month old. The VII variant segregated from the other variants on the F1 axis and was associated with both low severity (*p* = 0.025) and with the ethnicity-2 group, 56.5% of patients from this ethnicity-2 group were infected by the VII variant, but this represented only 46.4% of all samples harboring the VII protein (*p* = 0.006). No variant was specifically associated with the highest severity (*p* = 0.99) whereas the IIV variant was correlated with mild severity (*p* = 0.003).

### Spatiotemporal distribution of the virus populations

**Fig. 4:**
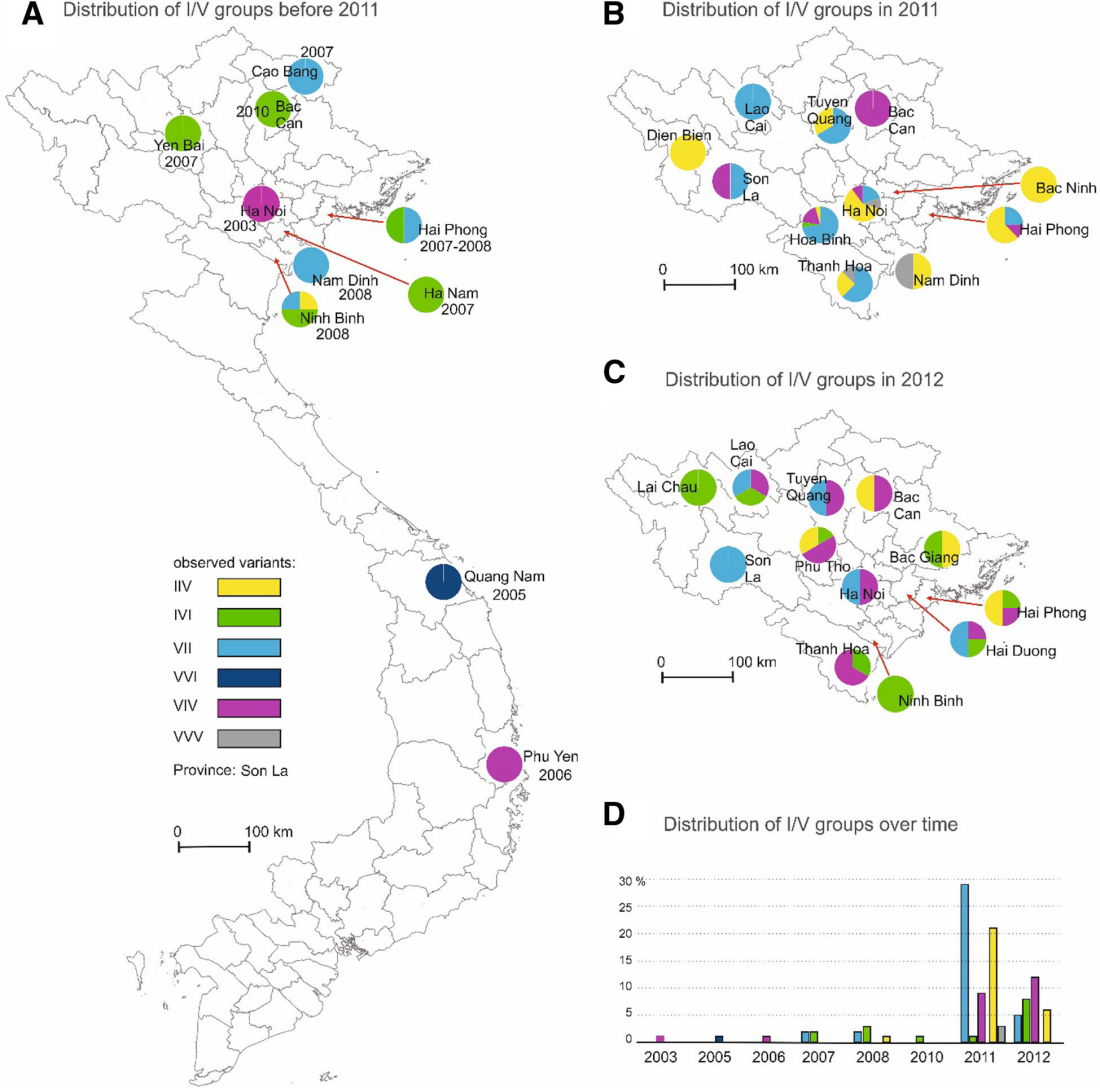
Spatiotemporal distribution of I/V variants. Color code: *Black*: VVV; *Light blue*: VII; *Yellow*: IIV; *Purple*: VIV; *Dark blue*: VVI

I/V variants present in the 2011 outbreak belonged mostly the IIV and VII populations which were already present in Northern Vietnam (Fig. 4a) The IIV population was previously detected in 2008 in the Ninh Binh province whereas VII variants were detected in Cao Bang and Hai Phong in 2007 and in Nam Dinh and Ninh Binh 2008. VII and IIV variants represented 46% and 33.3% of the samples collected in 2011, respectively (Fig. 4b, d). Other mutant populations detected in 2011 were: IVI (1.7%) already detected in 2007 in Yen Bai and Han Nam, in 2008 in Ninh Binh and in 2010 in Hai Phong and Bac Kan; VIV (14.3%) previously found in 2003 in Ha Noi and in 2006 in Phu Yen; and VVV (4.8%) (Fig. 4a). The VVV variants were not detected in samples collected prior to the 2011–212 outbreak. The mutant populations detected in 2012 were IIV and VII whose prevalence was reduced to 16.2% and 19.3% and VIV and IVI which prevalence rose to 38.7% and 25.8%, respectively (Fig. 4c, d). The VVV mutant was found only in 2011 in Thanh Hoa, Nam Dinh and Ha Noi (Fig. 4b, d). With respect to spatial distribution, the rise of variants VII and IIV observed in 2011 was not located in a specific area but covered most of the sampling sites (8 out of 11). The replacement of the IIV and VII variants by the IVI and VIV variants followed a similar pattern confirming the wide-spread diffusion of the outbreak. The number of sites with more than two variants was higher in 2011 than in 2012. The IIV variant was the most widely spread in 2011 but became the least widely spread in 2012 (Fig. 4b, c). Conversely, the IVI variant which was the least widely spread in 2011 and found in only one province, i.e. Hoa Binh, became the most widely spread in 2012. The VVV variant was found only in 2011 in three provinces, in the South Eastern part of North Vietnam each time in association with the IIV variant (Fig. 4b, c). The VVI variant was detected only in Quang Nam, Central Vietnam and prior to 2011.

## Discussion

This work provides an insight on the evolution and dynamics of the EV-A71 enterovirus during the first outbreak recorded in North Vietnam in 2011–2012. The first conclusion is that the 2011–2012 outbreak in North Vietnam was not due to a single exogenous strain imported from South Vietnam where HFMD outbreaks were present [19] or from another region. All variant populations observed during the 2011–2012 outbreak were already present in North Vietnam. The only exception is the VVV population which was found only in 2011 in three different provinces. However, the phylogenetic analysis indicated that this VVV variant was the closest to the root and therefore to the mother and oldest population. The reason for the lack of VVV variants in samples older than 2011 is most likely related to the low number of samples and to the low prevalence of this population. Furthermore, this 2011–2012 outbreak was also characterized by the cocirculation of the same four variant populations with a replacement between 2011 and 2012. The VII and IIV variants which were the most prevalent in 2011 were replaced by IVI and VIV populations in 2012. There is no clear explanation for the replacement of the main variant populations between 2011 and 2012 but it could be related to immunoresistance acquired during the first half of the outbreak in 2011 The surge of variants VII and IIV in the first part of the epidemic could not be related to any measured parameters and altogether the question remains of what triggered the outbreak in 2011 although all virus populations were already present. All I/V populations present at the beginning of the outbreak were capable of triggering it as shown by the replacement in 2012. It is not related either to the subgenogroup since the populations which emerged in 2012 belonged to two different subgenogroups, the VIV variant belonging the subgenogroup C4 and the IVI variant being a member of subgenogroup C5. A partial explanation could be a differential susceptibility of the human population which could have been slightly more susceptible to the VII and IIV groups. Another explanation might be found in the spatial distribution of the various variant groups, the socio-economic pattern and the route of dissemination. This work was not structured to address this issue and specific sampling schemes as well as transversal analyses should thus be further undertaken.

Another main outcome of this work is the observed correlation between I/V variant groups and phylogeny, pathogenicity and ethnicity. One hypothesis is that fixation of mutations in VP1 could be related to the VP1 function itself. Li et al. [16] reported virulent determinants in VP1 located at position 710 and 729 with Glutamic acid, Glycine or Arginine being associated to severe cases at position 710 and Glutamic acid at position 729 being also a marker of severity. In this study we didn’t see this correlation with position 710 bearing 90 Glutamic acid, 6 Glycine and 12 Glutamine and position 729 bearing 107 Aspartic acid and 1 Glycine both out of 108 sequences. These amino acids were found in strains associated to mild and moderately severe cases. No highly severe case was found in this work. I/V groups, although based on the relative arrangement of only three amino-acids, overlap the different clusters identified. These clusters correspond to genetically different populations characterized by specific polymorphism traits. This overlap between the specific combination of I/V residues at three positions and the phylogeny established on the nucleotide sequences suggests the occurrence of a selective pressure on the I/V arrangement. The high conservation of the proteins, despite variability at the nucleotide level, indicative of a negative, or purifying, selection pressure, indicates that the clustering at the protein level is driven by the I/V arrangement. The remaining question is what is the selective pressure acting on I/V variants and what could be the role of these I/V mutations. I/V mutations are located in the VP1 protein at positions 814, 827 and 849 of the polyprotein. The region from amino acid 697 to 862 on the EV-A71 polyprotein at the level of the VP1 protein was shown to be crucial for increasing the strength of protein-protein interactions in the capsid and its stability. This increased stability strongly enhances the pathogenicity and survival of the virus in the gastrointestinal track [15]. Isoleucine and valine are aliphatic hydrophobic amino acid mediating the core structure of the protein and have been reported to be involved in virulence and pathogenicity in several viruses, coxsackievirus B3 [25], Moloney murne Leukemia Virus [26], Infectious Bursal Disease virus [32], Japanese Encephalitis virus [30], and Simian-Human Immunodeficiency Virus [23]. The recurrent reports of the involvement of isoleucine and valine in the viral pathogenicity process in different viruses as well as their involvement in the selective pressure applied on the EV-A71 isolates analyzed in this work suggest that the I/V pattern at positions 249, 262 and 284 on the VP1 protein might indeed play a role in pathogenicity. The observed correlation of I/V variant populations with severity and ethnicity strengthen this hypothesis. However, the ethnicity correlation could be a result of spatial structuration since ethnicity-2 is mostly present in the Hòa Bình province. This in turn would suggest that the various EV-A71 variants display a geographic specificity.

## Conclusion

Altogether, these data suggest that EV-A71 strains could remain in a low level, asymptomatic state, in genomic stasis and with a geographic structuration. The cause for outbreaks should thus be sought for in the socio-economic patterns rather than in exogenous emergence. Further investigations are needed to investigate this hypothesis and to bring valuable information for the management of this major pediatric disease.

## Additional file

Additional file 1: Table S1. Severity levels of HFMD cases according to guidelines from the Vietnamese Ministry of Health. (DOCX 15 kb)
