# Supplementary material for: Valine/isoleucine variants drive selective pressure in the VP1 sequence of EV-A71 enteroviruses
Source: BMC Infect Dis. 2017 May 8;17:333. doi: 10.1186/s12879-017-2427-4 (PMC5422960; doi:10.1186/s12879-017-2427-4)
Supplement: Additional file 1: Table S1. — Severity levels of HFMD cases according to guidelines from the Vietnamese Ministry of Health. (DOCX 15 kb) [file 12879_2017_2427_MOESM1_ESM.docx]

**Table S1. Severity levels of HFMD cases according to guidelines from the Vietnamese Ministry of Health**

Level 1: Only mouth ulcers and/or skin lesions

Level 2a: have in addition one of any signs below:

- - Startled in history less than 2 times/30 min and no startled at examination.
  - Fever over 2 days or temperature of patient over 39^o^C, vomiting, insomniac, irritability

Level 2b**:** Have in additions signs from group 1 or group 2 below:

* Group 1: have one of any signs below

- - Startled when examination.
  - Startled in history ≥ 2 times /30 min.
  - Startled in history together with one sign below:
  - Drowse
  - Pulse > 130 times/min (child in the rest, no fever).

* Group 2: have one of any signs below

- - High fever ≥ 39,5^o^C (anal) and no response to fever release drug.
  - Pulse > 150 times /min (child in the rest, no fever).
  - Ataxia (locomotor ataxy), tremors
  - Nystagmus, squinting eye
  - Limb weakness of limb paralysis
  - Cranial nerve paralysis

Level 3: Have in addition the following signs/symptoms

- - Pulse > 170 time/min (child in the rest, no fever).
  - Some cases with slow pulse (very severe sign).
  - Sweating, coldness of partial or whole body.
  - Systolic blood pressure (SBP) increases
  - Child under 12 months of age: SBP > 100 mmHg.
  - Child from 12 to 24 months of age: SBP > 110 mmHg.
  - Child more than 24 months of age: SBP > 115 mmHg.
  - Respiratory abnormalities (tachypnea, abnormal breathing)
  - Perceptual disorders (dysaesthesia): Glasgow < 10 scores.
  - Increase muscle tone.

Level 4: have in addition, one of any signs below

- - Shock
  - Acute pulmonary edema
  - Cyanosis, SpO2 < 92%.
  - Suspension of breathing, breathing step

**Declarations**

**Ethics approval and consent to participate**

This work was conducted strictly following the requirements of the Vietnamese Ministry of Health and under the Law of Communicable Diseases Prevention and Control passed in 2007. This work was conducted under the control of NIHE Ethic committee. These procedures include a written agreement from parents or their legal representatives.

**Consent for publication**

Not Applicable

**Availability of data and material**

All data are publicly available and sequences have been deposited in Genbank. Accession numbers of sequences deposited in Genbank are ranging From KX906261 to KX906368 (108 sequences).

**Competing interests**

The authors declare that there is no competing interests

**Funding**

The work was supported by internal grants from NIHE and from DUKE/NUS for sequencing. NDN was in part supported by European Erasmus Mundus project MAHEVA and by the PEPS project MoDyCa from University of Montpellier and CNRS.

**Authors' contributions**

NDN participated to all parts of the work

OMS, YAH, RC and DJG generated all sequences

LTTH, LTSH, VDT and NTHT participated to sample collection and molecular analysis and amplification

AA designed all maps and spatiotemporal analysis

LG, CM, PR, GK, EC and RF participated to all bioinformatic, statistic and phylogenetic analyses

CD, NTH and TND have provided fruitful advises and discussions

RF supervised the work and participated to all analyses and to the writing

**Acknowledgements**

Not applicable
